# A One‐Structure‐Based Multieffects Coupled Nanogenerator for Simultaneously Scavenging Thermal, Solar, and Mechanical Energies

**DOI:** 10.1002/advs.201700622

**Published:** 2017-12-08

**Authors:** Yun Ji, Kewei Zhang, Ya Yang

**Affiliations:** ^1^ Beijing Institute of Nanoenergy and Nanosystems Chinese Academy of Sciences Beijing 100083 P. R. China; ^2^ CAS Center for Excellence in Nanoscience National Center for Nanoscience and Technology (NCNST) Beijing 100190 P. R. China; ^3^ University of Chinese Academy of Sciences Beijing 100049 P. R. China

**Keywords:** barium titanate, energy scavenging, multieffects, nanogenerators

## Abstract

Rapid advances in various energy harvesters impose the challenge on integrating them into one device structure with synergetic effects for full use of the available energies from the environment. Here, a multieffect coupled nanogenerator based on ferroelectric barium titanate is reported. It promotes the ability to simultaneously scavenging thermal, solar, and mechanical energies. By integration of a pyroelectric nanogenerator, a photovoltaic cell, and a triboelectric–piezoelectric nanogenerator in one structure with only two electrodes, multieffects interact with each other to alter the electric output, and a complementary power source with peak current of ≈1.5 µA, peak voltage of ≈7 V, and platform voltage of ≈6 V is successfully achieved. Compared with traditional hybridized nanogenerators with stacked architectures, the one‐structure‐based multieffects coupled nanogenerator is smaller, simpler, and less costly, showing prospective in practical applications and represents a new trend of all‐in‐one multiple energy scavenging.

Integration of various energy harvesters for scavenging multitype energies is recognized as one of the most important energy‐related technologies due to air pollution and insufficient fossil fuel supplies.^1^, ^2^, ^3^, ^4^, ^5^ To date, binary/multivariate hybridization among piezoelectric, triboelectric, pyroelectric, thermoelectric (TE), electromagnetic, and photovoltaic energy harvesters, either in series or in parallels, has been widely performed to realize self‐powered operation of electronics with high power consumption.^6^, ^7^, ^8^, ^9^, ^10^, ^11^, ^12^, ^13^ Despite significant advances in enhancement of overall power output, complex device structure limits the interaction of various effects since the generated electricity is separately exported through distinct electrodes. Recently, very few reports have demonstrated the design of one‐structure‐based hybridized device with only a pair of electrodes,^14^, ^15^, ^16^ which is highly desirable for achieving synergetic promotion and represents a new trend of all‐in‐one multiple energy scavenging. The core of such device is multifunctional material beneficial for combining multieffects within one single device. Barium titanate (BaTiO_3_, signed as BTO), a ferroelectric material at room temperature with high piezoelectric response and dielectric constant,^17^ offers a unique opportunity for such an investigation. As is well‐known, BTO carries a switchable spontaneous polarization, making it a satisfying piezoelectric and pyroelectric material.^18^, ^19^, ^20^ With direct band gap of ≈3.3 eV,^21^ this material can also absorb photons to produce a steady‐state photocurrent under the bulk photovoltaic effect.^22^, ^23^, ^24^

Taking advantage of multifunctional BTO, we design a coupled nanogenerator combining pyro‐tribo‐piezo‐photoelectric effects in one structure with only two electrodes, as principally depicted in **Figure** **1**a. The component of pyroelectric nanogenerator (PENG) can scavenge the thermal energy from temperature oscillation by the pyroelectric effect, the component of photovoltaic cell (PVC) can scavenge the solar energy from light illumination by the bulk photovoltaic effect, and the component of triboelectric–piezoelectric nanogenerator (TPiENG) can scavenge the mechanical energy from strain‐induced deformation of BTO as well as triboelectrification between nylon and fluorinated ethylene propylene (FEP) films by the piezoelectric effect and triboelectric effect. Multieffects coexist in one structure and interact with each other to alter the electric output, promoting the ability to individually/simultaneously scavenge multitype energies whenever and wherever one or all of the energy resources are available. Compared with traditional hybridized nanogenerators with stacked architectures, the one‐structure‐based multieffects coupled nanogenerator is simpler, smaller, and less costly. Finally, a complementary power source with peak current of ≈1.5 µA, peak voltage of ≈7 V, and platform voltage of ≈6 V is successfully achieved by the multieffects coupled nanogenerator.

**Figure 1:**
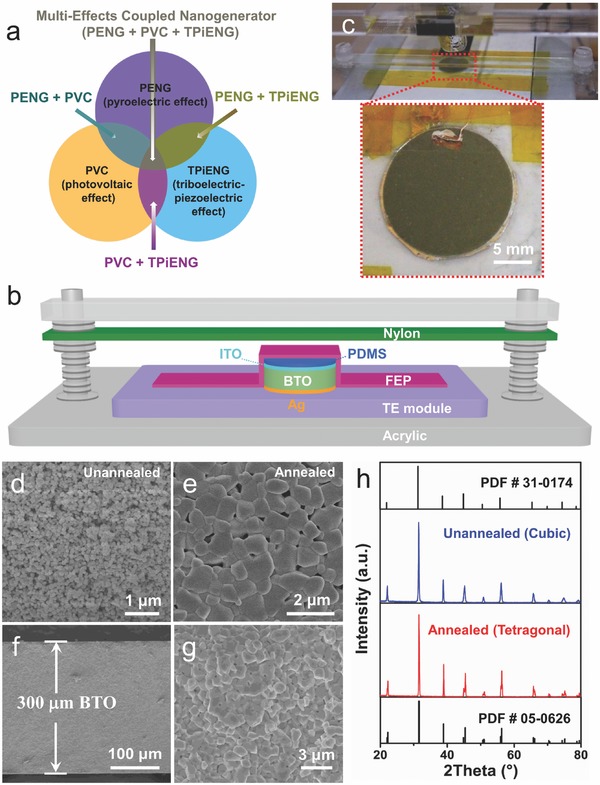
Design and structure of multieffects coupled nanogenerator. a) Design principle of coupled nanogenerator. b) Illustration of a coupled nanogenerator combining piezo‐tribo‐pyro‐photoelectric effects. c) A photo image of the fabricated device using multifunctional BTO as core component. SEM images of d) the raw BTO powders, e) the BTO powders after annealing at 1473 K, f) cross‐sectional view of the prepared BTO ceramic disk, and g) enlarged view of the prepared BTO ceramic disk. h) XRD patterns of BTO powders, showing that the BTO transforms from cubic phase to tetragonal phase after annealing.

The coupled nanogenerator consists of multilayers as schematically illustrated in Figure 1b. The deposited indium tin oxide (ITO) and silver (Ag) films act as the top and bottom electrodes. The ferroelectric BTO serves as the piezoelectric, pyroelectric, and photoelectric material. The nylon film works as flexible vibrating film to provide external strain on the BTO ceramic disk, as well as create single‐electrode triboelectrification with the FEP film. The polydimethylsiloxane (PDMS) works as a protecting layer to sustain the conformation of ITO/BTO/Ag. The TE module serves as the source of temperature oscillation. By constructing all the above layers together, the coupled nanogenerator functionally possesses a PENG, a PVC, and a TPiENG, sharing one pair of electrodes. Figure 1c shows a photo image of the final device with BTO ceramic disk (16.8 mm in diameter) as the core component. The ferroelectric BTO ceramic disk was fabricated by dry‐pressing BTO powders. As shown in Figure 1d, the BTO powders demonstrate uniform spherical morphology with diameter of ≈150 nm. After annealed at 1473 K for 2 h, the nanoscale BTO powders agglomerate into microscale particles with the size of ≈0.5–1.5 µm (Figure 1e), indicating that the annealing temperature of 1473 K is suitable for acquiring high‐performance BTO ceramic. The fabricated BTO ceramic disk is about 0.3 mm in thickness, as shown in the cross‐sectional scanning electron microscopy (SEM) image in Figure 1f. The high‐magnification SEM image in Figure 1g confirms that the obtained BTO ceramic disk possesses high density across the entire volume. Figure 1h shows the X‐ray diffraction (XRD) patterns of the BTO powders before and after annealing, where the single peak at 2θ = 45° is split into two peaks at 2θ = 44.8° and 2θ = 45.4°, suggesting the crystalline structure transforms from cubic phase (PDF # 31‐0174) to tetragonal phase (PDF # 05‐0626) at the given annealing temperature. This result indicates the ferroelectric nature of the fabricated BTO ceramic disk, since BTO is ferroelectric when tetragonal, but paraelectric when cubic.^25^

The typical electrical outputs of individual nanogenerators are shown in **Figure** **2**. For the individual PENG, a periodic change of temperature from 302 to 307 K with peak heating rate of 0.34 K s^−1^ was applied on the device by a commercial TE module (Figure S1, Supporting Information). As shown in Figure 2a, a sharp positive current/voltage peak (15.2 nA/1.5 V) is produced when the temperature is quickly increased from 302 to 307 K, and then returns to zero when there is no temperature change. An equivalent negative current/voltage peak is observed when the temperature is recovered back to 302 K. To be noted, the recovery of voltage is slower than that of the current, thus we prolonged the interval time of temperature oscillation from 3 to 6 min. After reversely connecting the device to the measurement systems, opposite output signals are observed (Figure S2, Supporting Information), indicating that the measured signals are produced by the pyroelectric effect. Figure 2b presents the dependence of output current and the corresponding instantaneous power on external loading resistance. Owing to the ohmic loss, the peak value of output current decreases as the resistance increases. Consequently, the output power reaches maximum value of 7.8 nW at the matched loading resistance of 150 MΩ, that is, the internal resistance of the PENG is 150 MΩ. On the other hand, reverse current/voltage signals are observed when a periodic change of temperature from 302 to 298 K was applied on the device (Figure S3, Supporting Information). The electrical outputs of individual PVC were measured under light‐emitting diode (LED) illumination (λ = 405 nm). As demonstrated in Figure 2c, obvious two‐stage current signals are observed. The sharp peak of 40.5 nA in the first stage is induced by the photovoltaic effect coupled with the illumination‐induced pyroelectric effect. The stable platform of 10.1 nA in the second stage is thus considered to the net photovoltaic effect, since the illumination‐induced heat effect gradually disappears. Similarly, the output voltage reaches a peak value of 0.8 V and then slightly decreases to a stable value of 0.6 V. Maximum output power of 7.6 nW is obtained at a matched loading resistance of 25 MΩ (Figure 2d), suggesting a lower internal resistance of the PVC as compared to the above PENG. Besides, the performance of ferroelectric PVC can be further enhanced by regulating the depolarization field.^26^, ^27^ To quantitatively characterize the performance of individual TPiENG, an airflow of 15 m s^−1^ is generated by a commercial air blower with a distance of 12 cm away from the device. As shown in Figure 2e, the short‐circuit current of the TPiENG has a continuous AC‐type output at an amplitude of ≈3.5 µA, and the signals follow the oscillation of the nylon film. Normally, the piezoelectric and triboelectric devices deliver high voltages. Here, it is noticed that the baseline of voltage curve is swinging and the voltage value of the TPiENG is difficult to be distinctly detected (<0.1 V), which is likely attributed to the cancellation between the piezoelectric and triboelectric effects.^5^, ^28^ To distinguish the contribution of piezoelectric nanogenerator (PiENG) on the obtained current signals of TPiENG, a comparative experiment was conducted by replacing the FEP film with the nylon film to avoid the triboelectrification. As shown in Figure S4 (Supporting Information), the PiENG can deliver an output current of ≈1.1 µA. Once an external loading resistance is applied, the amplitude of the output current drops as the resistance increases, as shown in Figure 2f. At the matched loading resistance of 4 KΩ, the instantaneous output power reaches 11.8 nW at an airflow speed of 15 m s^−1^. The performance of airflow‐driven TPiENG could be further enhanced through geometric optimization.^29^, ^30^

**Figure 2:**
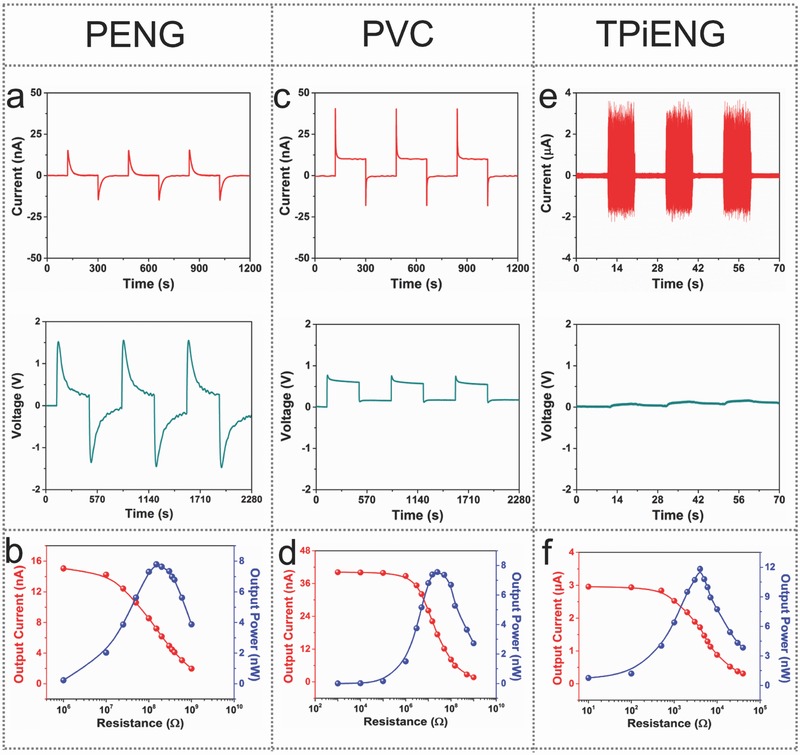
Electrical output of individual PENG, PVC, and TPiENG. a,c,e) Short‐circuit current and open‐circuit voltage. b,d,f) Dependence of output current and the corresponding instantaneous power on external loading resistance.

The observed pyroelectric ability of the device was further investigated under a series of cooling and heating conditions. As seen from **Figure** **3**a–d, positive heating/cooling rate (d*T*/d*t*) leads to positive current/voltage signals while negative d*T*/d*t* causes negative signals. Besides, the characteristic of current/voltage curve is in accordance with the characteristic of d*T*/d*t*. Figure 3e clarifies the measured current/voltage peaks as a function of d*T*/d*t*, exhibiting a near‐linear dependency. On increasing d*T*/d*t* from −0.85 to 0.98 K s^−1^, the peak current increases from −44.4 to 49.8 nA, and the peak voltage increases from −4.6 to 2.9 V. According to our previous work,^5^ the output performance of PENG could be further enhanced by increasing the frequency of temperature oscillation. Normally, the differential change of temperature‐dependent spontaneous polarization is defined as the pyroelectric coefficient (*p*). By using the active area (*A*) of BTO ceramic disk (≈2.22 cm^2^), the pyroelectric coefficient of BTO can be calculated from the equation of *I* = *pA*(d*T*/d*t*). The calculated value is about 22.5–25.9 nC cm^−2^ K^−1^, as presented in Figure 3f.

**Figure 3:**
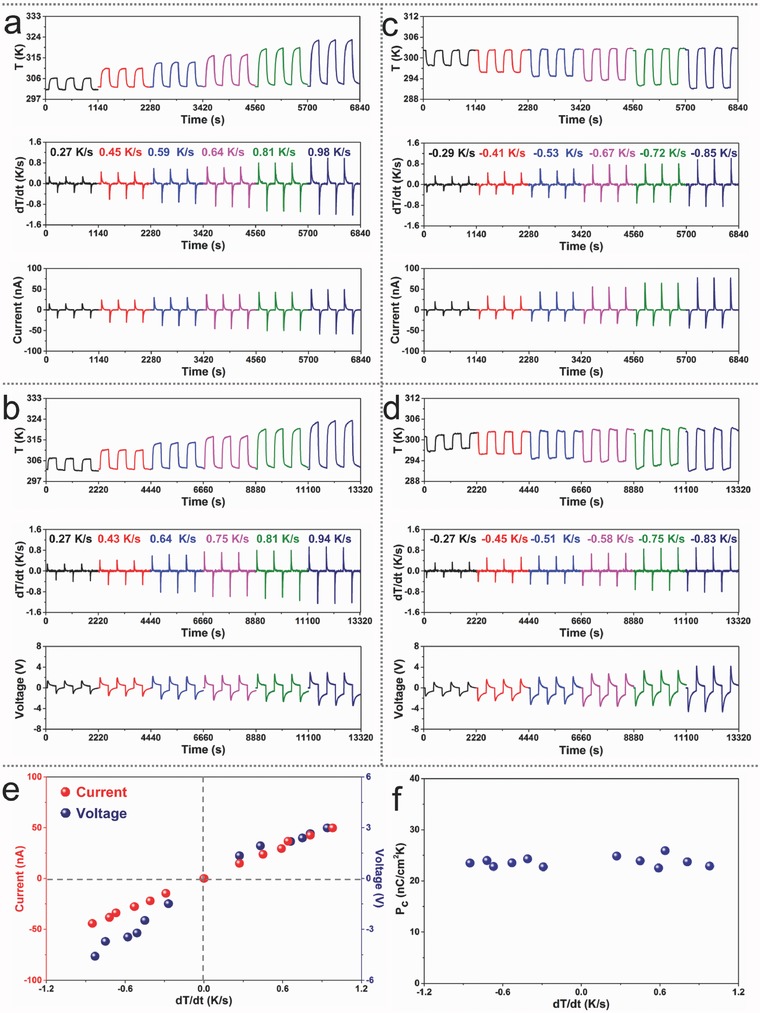
Temperature‐dependent output characteristics of PENG. The periodic changes of temperature, the corresponding differential curves, output currents, and output voltages under periodic a,b) heating condition and c,d) cooling condition. e) Dependence of output current and voltage on the heating/cooling rate. f) Calculated pyroelectric coefficient based on current values in (e).

The coupling effect between PENG and PVC was measured by simultaneously cooling/heating and lighting the device. **Figure** **4**a shows representative current curves of the “PENG + PVC” under cooling and lighting condition as compared with that of the individual units. The individual PVC delivers a peak current value of 40.5 nA with a platform value of 10.1 nA (*I*_2_) under 405 nm LED illumination. After synchronously beginning the cooling and lighting, the “PENG + PVC” delivers a peak current value of −44.3 nA with a platform value of 11.8 nA (*I*_3_). It can be seen that the coupling effect promotes the platform current value of PVC by nearly 16.8%. On the other hand, under heating condition (0.98 K s^−1^), the peak current of the “PENG + PVC” reaches as high as 92.9 nA (Figure 4b), corresponding to an increase ratio of 86.5% as compared with the peak current of individual PENG (49.8 nA). The current curves of “PENG + PVC” under 405 nm LED illumination at different cooling/heating rate are shown in Figure S5 (Supporting Information). For an accurate comparison, the platform currents are extracted and summarized in Figure 4c, which reveals a proportional relationship between *I*_3_ and d*T/*d*t*. Besides, we further evaluate the total transferred charges of the coupled “PENG + PVC” and the individual units by integrating the current curves over the time interval of 6 min, as shown in Figure 4d. It can be found that the PVC can provide a constant charge amount of ≈35.3 nC, while the total charge amount of PENG increases linearly with d*T/*d*t*. It deserves to be mentioned that the slope of heating process (right side of Figure 4d) is slightly smaller than that of the cooling process (left side of Figure 4d), which may be attributed to the weaker heat transmission. After coupling PENG and PVC together, the transferred charge of “PENG + PVC” is larger than that of the individual PVC as well as the individual PENG. Under 405 nm LED illumination, the positive peak voltage of “PENG + PVC” reaches as high as 1.6 and 4.2 V at heating rate of 0.98 K s^−1^ and cooling rate of −0.85 K s^−1^, respectively, as depicted in Figure S6 (Supporting Information).

**Figure 4:**
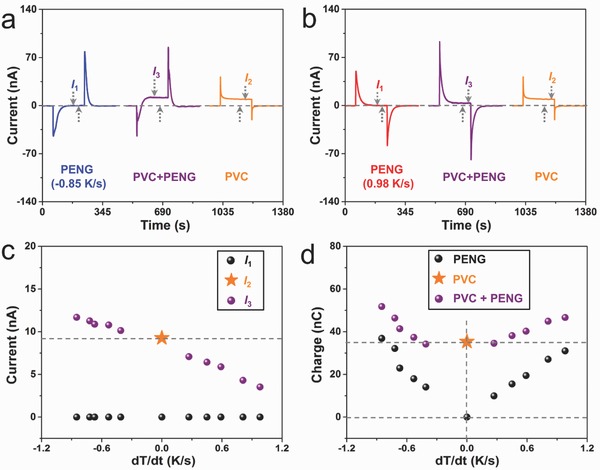
Coupling effect between PENG and PVC. Representative current curve of the “PENG + PVC” under a) cooling condition and b) heating condition as compared with that of individual units. c) Platform current of the device as a function of the heating/cooling rate. d) Transferred charges of “PENG + PVC” and individual units at different heating/cooling rate.

A commercial full‐wave bridge rectifier (DF 06, Frontier Electronics) was connected to the device to convert the AC‐type output into DC‐type output. As demonstrated in **Figure** **5**a–c, the peak current is ≈1.48 µA, ≈14 nA, and ≈40 nA for the TPiENG, PVC ,and PENG, respectively. The rectified peak voltage of PENG under heating rate of 0.98 K s^−1^ is ≈2.75 V, as shown in Figure S7 (Supporting Information). For the coupled nanogenerator of “PENG + PVC + TPiENG,” the peak current is slightly larger than that of the TPiENG, and the coupling characteristic can be clearly observed from the enlarged view of the current profile at the beginning of airflow (Figure S8, Supporting Information). By integration of PVC, PENG, and TPiENG into one device with only two output electrodes, a complementary power source with peak current of ≈1.5 µA, peak voltage of ≈7 V, and platform voltage of ≈6 V is successfully achieved (Figure S8, Supporting Information). To evaluate the performance of coupled nanogenerator as an uninterrupted power, a 0.33 µF capacitor was used for temporary charge storage. Figure 5d displays the charging performance of the capacitor charged by the coupled nanogenerator of “PENG + PVC + TPiENG” as compared with the individual units. It is obvious that the capacitor could be charged faster by the coupled output than that by only one individual output. The voltage of capacitor reaches up to 1.1 V in 10 s by the coupled nanogenerator of “PENG + PVC + TPiENG” under heating rate of 0.98 K s^−1^, 405 nm LED illumination and airflow speed of 15 m s^−1^.

**Figure 5:**
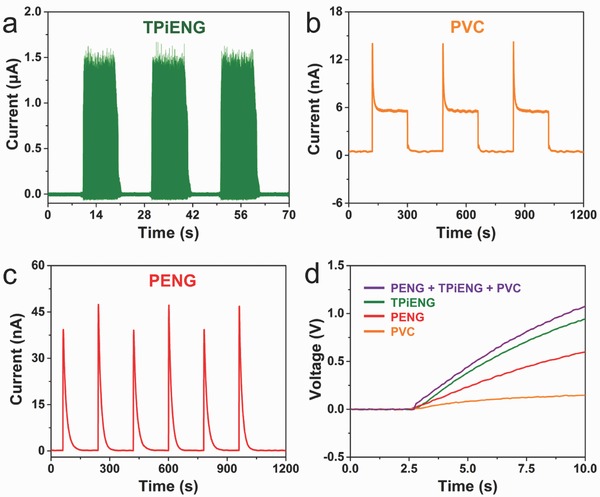
Rectified output current and charging performance. Rectified output current of a) TPiENG at airflow speed of 15 m s^−1^, b) PVC under 405 nm LED illumination, and c) PENG under heating rate of 0.98 K s^−1^. d) Measured voltage curves of a 0.33 μF capacitor charged with the coupled nanogenerator of “PENG + PVC + TPiENG” as compared with the individual units.

In summary, we have demonstrated a multieffects coupled nanogenerator based on ferroelectric BTO, which integrates a PENG, a PVC, and a TPiENG into a single structure with only two electrodes. Multieffects of the pyroelectric effect, the bulk photovoltaic effect, the piezoelectric effect and triboelectric effect coexist in one structure and interact with each other to alter the electric output, promoting the ability to individually/simultaneously scavenge thermal, solar, and mechanical energies whenever and wherever one or all of the energy resources are available. When all the PENG, PVC, and TPiENG are simultaneously working, the multieffects coupled nanogenerator exhibits a much better charging performance than that of the individual units (TPiENG, PVC, or PENG). Under heating rate of 0.98 K s^−1^, 405 nm LED illumination and airflow speed of 15 m s^−1^, a complementary power source with peak current of ≈1.5 µA, peak voltage of ≈7 V, and platform voltage of ≈6 V is achieved, which can charge a 0.33 µF capacitor easily to 1.1 V in 10 s. This work demonstrates the feasibility of ferroelectric BTO for multieffects coupled nanogenerator, which is prospective for maximizing energy scavenging from our surrounding environments.

## Experimental Section

*Materials*: BTO nanoparticles were purchased from Sinopharm Chemical Reagent Co. Ltd. Polyvinyl alcohol (PVA) was purchased from Sigma‐Aldrich Co. LLC. All chemicals were used without further purification.

*Preparation of ITO/BTO/Ag Ceramic Disk*: The BTO ceramic disk was prepared using a normal dry press process. Briefly, BaTiO_3_ powders were thoroughly mixed with several drops of PVA solution (2 wt%). The mixture was then dry‐pressed at 4 MPa into as‐prepared disk with 20 mm in diameter and 1 mm in thickness. Subsequently, the as‐prepared disk was heated up to 923 K for 1 h to eliminate the PVA binder, and then annealed at 1473 K for 2 h to form BTO ceramic disk. After sputtering silver (Ag) electrodes on the top and bottom side, the BTO ceramic disk was polarized under an electric field of 3.5 kV mm^−1^ for 30 min in silicon oil at room temperature. The applied electric field directed from the top side to the bottom side of the BTO ceramic disk. Finally, the poled BTO ceramic disk was top‐polished to a thickness of 0.3 mm, and then deposited with ITO by radio frequency magnetron sputtering as the top electrode. During ITO deposition, the sputtering power was set as 150 W, the working pressure was 0.3 Pa, and the argon (99.999% purity) flow rate was 80 sccm. The piezoelectric coefficient (*d*_33_) of the poled BTO ceramic disk was measured to be ≈180 pC N^−1^.

*Fabrication of Coupled Nanogenerator*: A commercial Peltier TE module with size of 120 × 80 mm^2^ (C1206, SINHEA) supported by a radiator was located in an acrylic frame. At the center of TE module, a piece of aluminum (Al) tape was affixed as bottom electrode of the device. Subsequently, the ITO/BTO/Ag ceramic disk was fixed on the TE module using conductive Ag paint (Leitsilber 200, Ted Pella, Inc.) with Ag side of the ceramic disk contacting the Al tape. A copper wire was fixed on upper side of the ceramic disk using Ag paste as top electrode of the device. The ceramic disk was further covered with PDMS as protective layer to sustain the conformation of ITO/BTO/Ag. A layer of adhesive FEP film (Witlan Industrial Co. Ltd.) was attached on the top of the PDMS layer as one of the triboelectric materials. Then, a layer of nylon film (145 × 10 × 0.06 mm^3^) which was used as the other triboelectric material as well as the vibration film was stringed together with an acrylic sheet and fixed on the supporting acrylic frame in sequence through a screw at each end. The distance between the nylon film and the FEP film as well as the distance between the nylon and the acrylic sheet was optimized to be 2 mm for maximizing the output performance of the TPiENG. Herein, the active area of the coupled nanogenerator is equivalent to the area of BTO ceramic disk that is ≈2.22 cm^2^.

*Characterization and Measurements*: SEM images were obtained with a Hitachi SU8020 instrument operated at 10.0 kV. XRD patterns of the BaTiO_3_ powders were recorded on a Philips X'Pert^3^ Powder X‐ray diffractometer with copper Kα radiation (λ = 1.54 Å). Temperature profiles of the device upon cooling/heating operation were recorded by an Optris PI400 IR thermographic camera. The 405 nm LED illumination is generated by a Zolix LED light source, keeping 2.3 cm away from the device. Output current and voltage signals of the device were measured by a Stanford Research SR570 low‐noise current preamplifier and a Keithley 6514 Electrometer, respectively.

## Conflict of Interest

*The authors declare no conflict of interest*.

## Supporting information

Supplementary
