# Supplementary material for: A One‐Structure‐Based Multieffects Coupled Nanogenerator for Simultaneously Scavenging Thermal, Solar, and Mechanical Energies
Source: Adv Sci (Weinh). 2017 Dec 8;5(2):1700622. doi: 10.1002/advs.201700622 (PMC5826984; doi:10.1002/advs.201700622)
Supplement: Supplementary file 1 — Supplementary [file ADVS-5-1700622-s001.pdf]

Copyright WILEY-VCH Verlag GmbH & Co. KGaA, 69469 Weinheim, Germany, 2017.

ADVANCED SCIENCE
Open Access

Supporting Information

for *Adv. Sci.,* DOI: 10.1002/advs.201700622

A One-Structure-Based Multieffects Coupled Nanogenerator for Simultaneously Scavenging Thermal, Solar, and Mechanical Energies

*Yun Ji, Kewei Zhang, and Ya Yang**

Supporting Information

A One-Structure-Based Multi-Effects Coupled Nanogenerator for Simultaneously Scavenging Thermal, Solar, and Mechanical Energies

Yun Ji, Kewei Zhang, and Ya Yang*

Y. Ji, Dr. K. Zhang, Prof. Y. Yang, Beijing Institute of Nanoenergy and Nanosystems, Chinese Academy of Sciences, Beijing 100083, P. R. China
Y. Ji, Dr. K. Zhang, Prof. Y. Yang, CAS Center for Excellence in Nanoscience, National Center for Nanoscience and Technology (NCNST), Beijing 100190, P. R. China
Y. Ji, University of Chinese Academy of Sciences, Beijing 100083, P. R. China
Y. J. and K. Z. contributed equally to this work.
E-mail: yayang@binn.cas.cn

Keywords: nanogenerators, barium titanate, multi-effects, energy scavenging

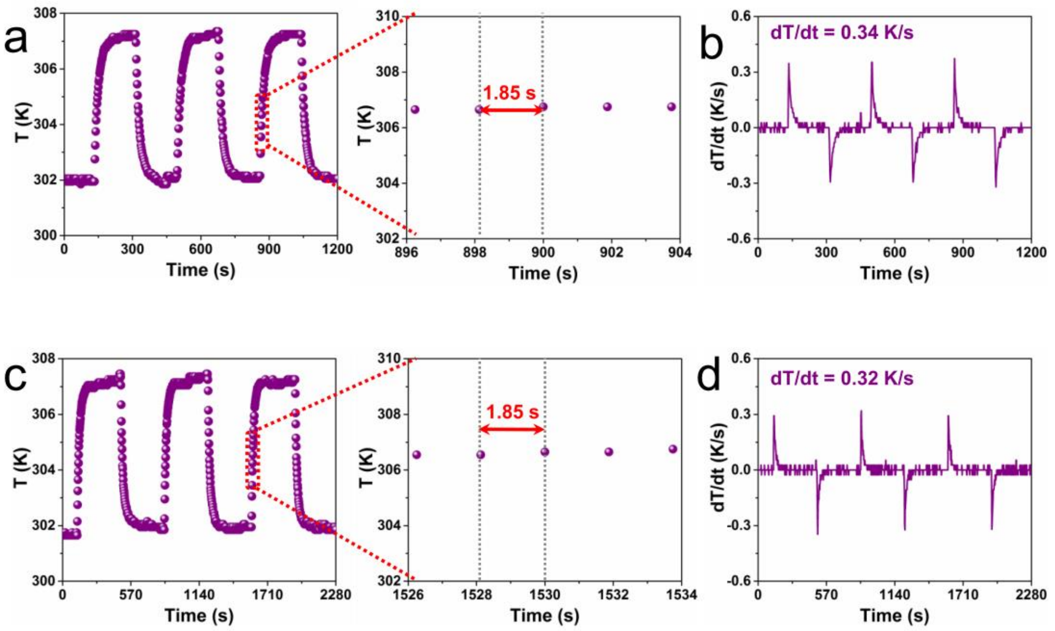

Figure S1. Periodic changes of temperatures and corresponding differential curves. a) Periodic changes of temperatures under heating condition with interval time of 6 min. b) The corresponding differential curves under heating condition with interval time of 6 min. c) Periodic changes of temperatures under heating condition with interval time of 12 min. d) The corresponding differential curves under heating condition with interval time of 12 min.

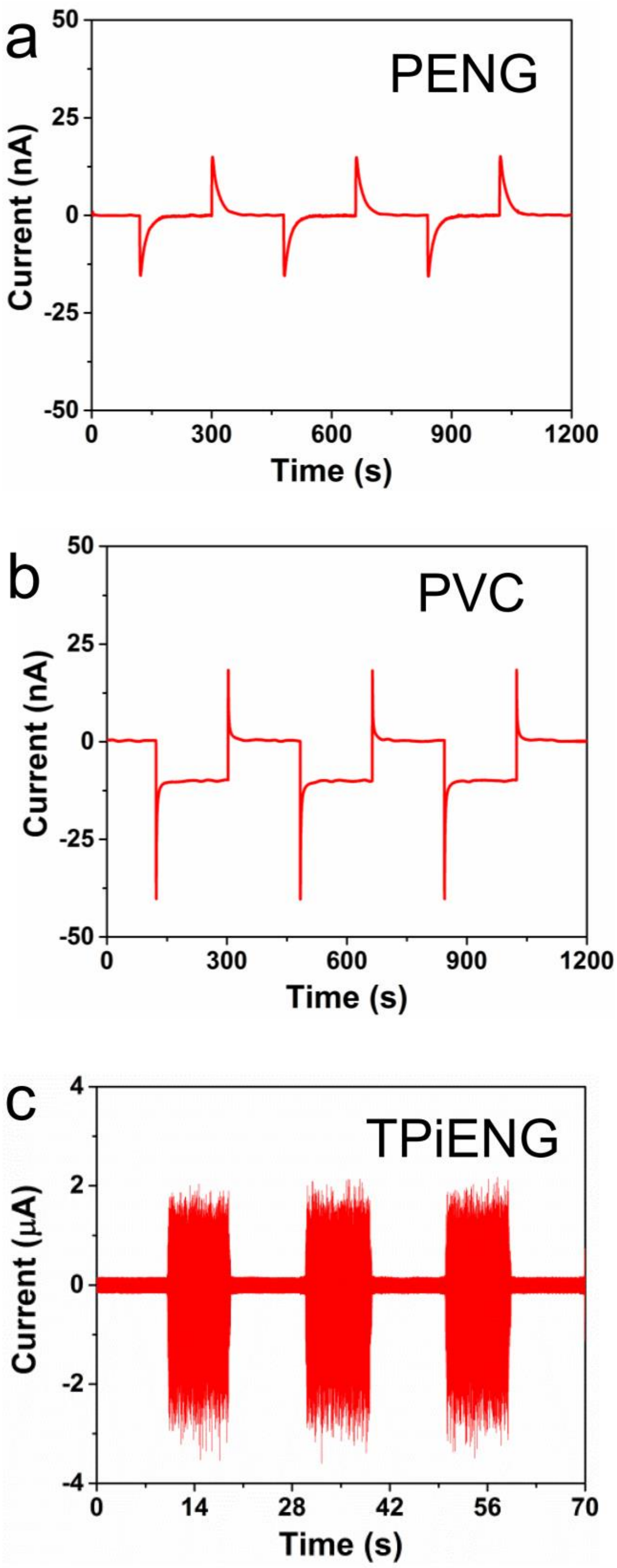

Figure S2. Output currents of individual PENG, PVC and TPiENG after reversing the connections: a) PENG, b) PVC, c) TPiENG.

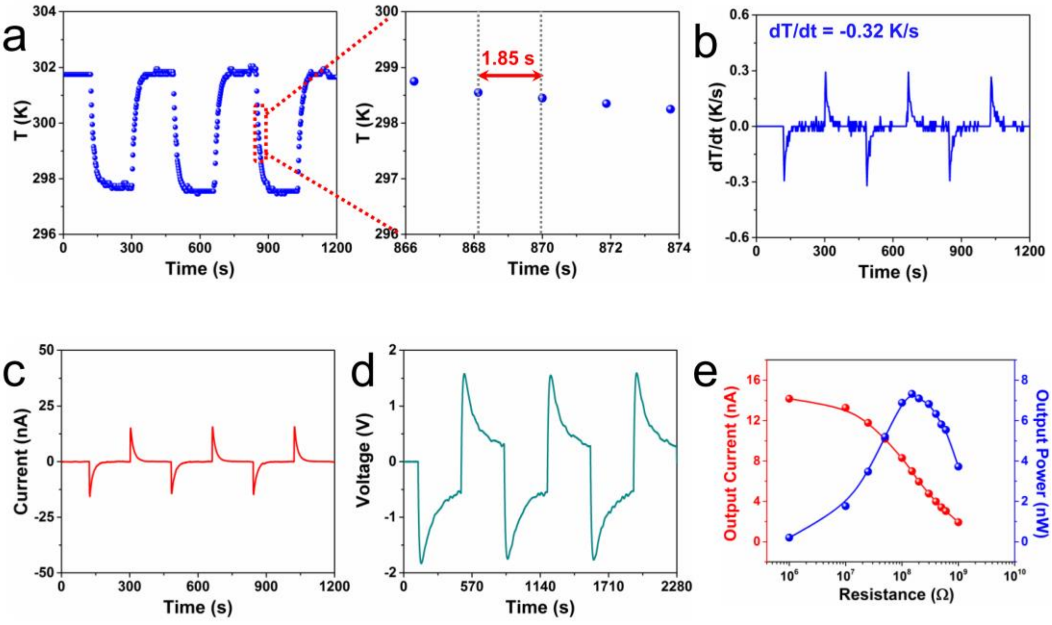

Figure S3. Electric output of the PENG under cooling rate of -0.32 K/s. a) Periodic changes of temperatures under cooling condition. b) The corresponding differential curves under cooling condition. c) Output current of the PENG. d) Output voltage of the PENG. e) Dependence of output current and the corresponding instantaneous power on external loading resistance.

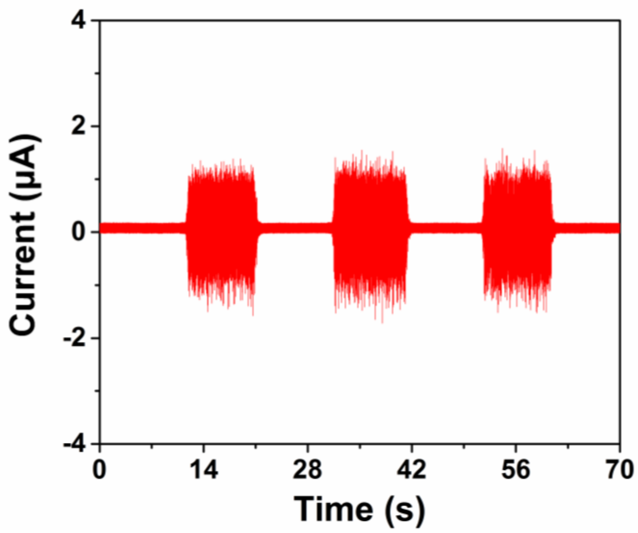

Figure S4. Short-circuit current of individual PiENG.

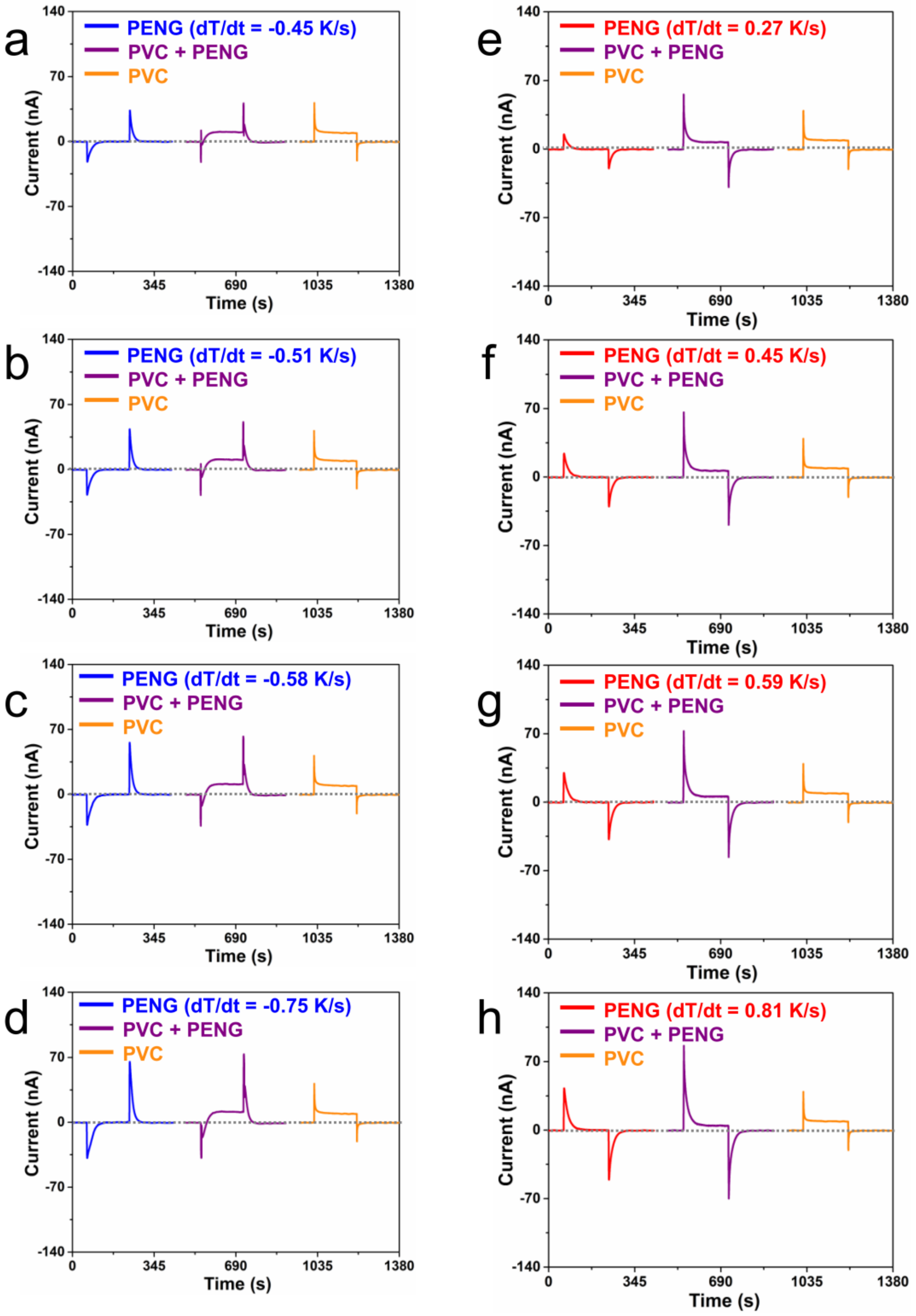

Figure S5. Output currents of the "PENG+ PVC" under different cooling/heating rates. a) dT/dt = -0.45 K/s, b) dT/dt = -0.51 K/s, c) dT/dt = -0.58 K/s, d) dT/dt = -0.75 K/s, e) dT/dt = 0.27 K/s, f) dT/dt = 0.45 K/s, g) dT/dt = 0.59 K/s, h) dT/dt = 0.81 K/s.

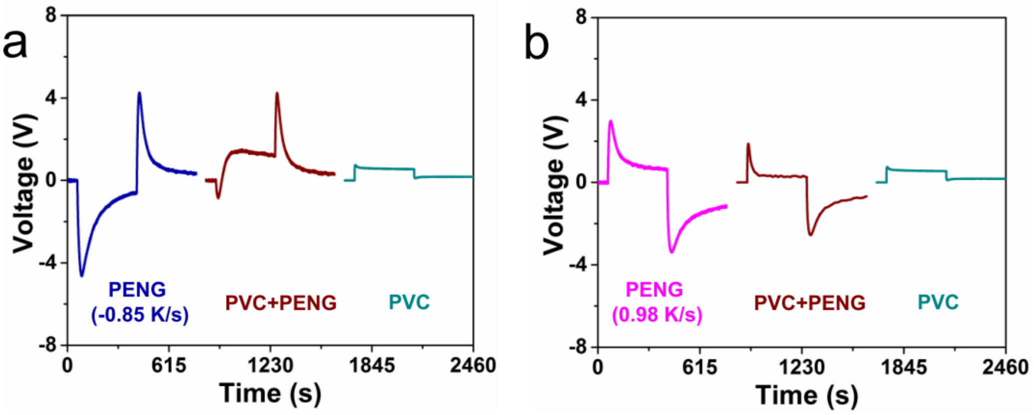

Figure S6. Output voltages of the "PENG + PVC" under -0.85 K/s and 0.98 K/s. a) Output voltage of the "PENG + PVC" under cooling rate of -0.85 K/s. b) Output voltage of the "PENG + PVC" under heating rate of 0.98 K/s.

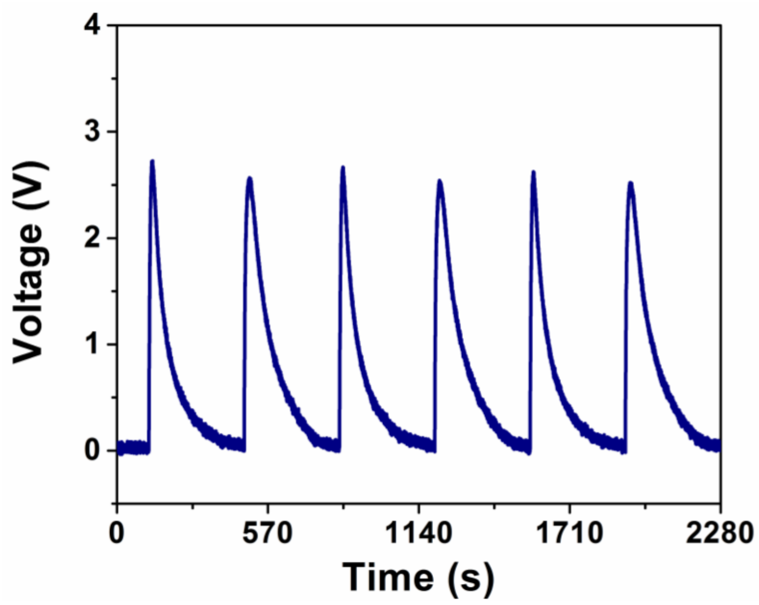

Figure S7. Rectified output voltage of the PENG under heating rate of 0.98 K/s.

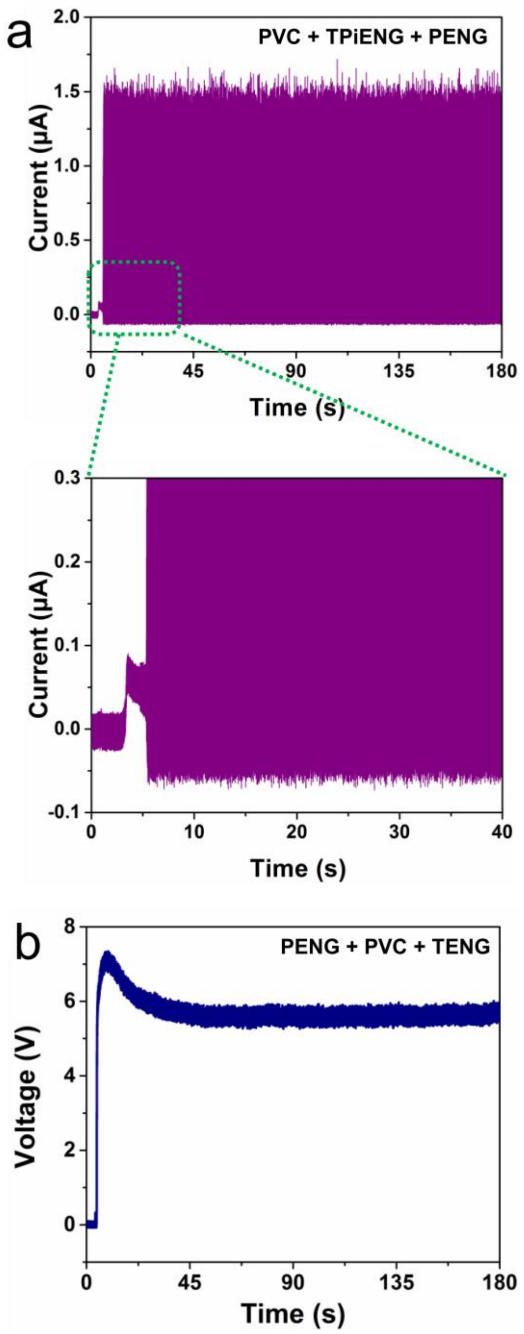

Figure S8. Rectified output current and voltage of the "PENG + PVC + TPiENG". a) Rectified output current of the "PENG + PVC + TPiENG" under heating rate of 0.98 K/s, 405 nm LED illumination, and airflow speed of 15 m/s. b) Rectified output voltage of the "PENG + PVC + TPiENG" under heating rate of 0.98 K/s, 405 nm LED illumination, and airflow speed of 15 m/s.
